# ^18^F-fluorodeoxyglucose positron emission tomography/computed tomography findings in descending necrotizing mediastinitis and cervical vertebral osteomyelitis in a cancer patient: A case report

**DOI:** 10.1097/MD.0000000000021353

**Published:** 2020-07-24

**Authors:** Jin Young Yoo, Min Young Yoo, Ki Hyeong Lee, Sung-Soo Koong

**Affiliations:** a Department of Radiology; b Department of Nuclear Medicine; c Department of Internal medicine, Chungbuk National University Hospital, Cheongju, Korea.

**Keywords:** 18F-FDG positron emission tomography, deep neck infection, descending necrotizing mediastinitis, spinal osteomyelitis

## Abstract

**Rationale:**

A deep neck infection (DNI) with descending necrotizing mediastinitis (DNM) has great clinical importance because of its high morbidity and mortality, particularly when associated with predisposing underlying disease. With the expanding clinical use of ^18^F-fluorodeoxyglucose (FDG) positron emission tomography/computed tomography (PET/CT), it may be necessary to perform FDG PET/CT for immediate diagnosis and treatment of DNM. To the best of our knowledge, this is the first case report of DNI with DNM diagnosed based on FDG PET/CT findings.

**Patient concerns:**

A 65-year-old man who underwent chemotherapy for stage IV lung cancer complained of sore throat, fever, and mild pain in the right upper arm for 4 days before admission.

**Diagnoses:**

FDG PET/CT revealed retropharyngeal abscess with acute osteomyelitis of the vertebral bodies of C4 and C5 and DNM. In blood and sputum cultures, *Klebsiella pneumoniae* was isolated. DNI with DNM was diagnosed based on contrast-enhanced neck and chest CT.

**Interventions and outcome:**

Because of his underlying condition, antibiotic therapy with ceftriaxone and ciprofloxacin was started. There was initial improvement, but the patient died after 2 weeks from sepsis and multiorgan failure.

**Lessons:**

The findings of DNI with DNM on FDG PET/CT were as follows: as an acute infection, DNM showed more severe uptake relative to the average maximum standardized uptake value of brown fat or physiologic muscle; showed the prevertebral uptake pattern rather than the paravertebral uptake pattern of brown fat; and showed continuous patterns of hypermetabolic lesions from the retropharyngeal/parapharyngeal space to the thoracic prevertebral space.

## 1 Introduction

Deep neck infections (DNIs) are uncommon in adults and mostly result from traumatic rupture of the pharyngeal mucous membrane. Nontraumatic DNIs are extremely rare in adults and mostly develop in immunocompromised patients.^[1,2]^ Because of the possible serious life-threatening complications, early diagnosis and management of DNI have great clinical importance.

Herein, we report a rare case of nontraumatic DNI with descending necrotizing mediastinitis (DNM) in a patient with a history of non-small cell lung cancer. To the best of our knowledge, this is the first case report of DNI with DNM detected on ^18^F-fluorodeoxyglucose (FDG) positron emission tomography/computed tomography (PET/CT). The patient has provided informed consent for publication of the case.

## 2 Case report

A 65-year-old man was admitted to our hospital for acute azotemia after 3 days of treatment with nonsteroidal anti-inflammatory drugs for sore throat. He was undergoing chemotherapy for stage IV non-small cell lung cancer without any evidence of residual viable malignancy. He complained of sore throat and mild pain in the right upper arm. Laboratory findings showed mild leukocytosis with the C-reactive protein elevated to 14.6 mg/L. The creatinine level was 4.0 mg/dL because of acute azotemia. At admission, his body temperature was 36.6°C, and his physical examination was unremarkable. No abnormalities were detected on chest radiography. In the nasal swab, the influenza A virus was confirmed. During the treatment of influenza A with talniflumate, he developed fever as high as 38.6°C and complained of aggravating pain and weakness in the right upper arm. He had no history of trauma or surgery in the head or neck region. After the blood culture, an empirical antibiotic therapy with intravenous ceftriaxone was started. Magnetic resonance imaging (MRI) of the brain revealed no metastatic or ischemic lesions. FDG PET/CT was performed to investigate bone metastases causing the right upper arm pain.

**Figure 1:**
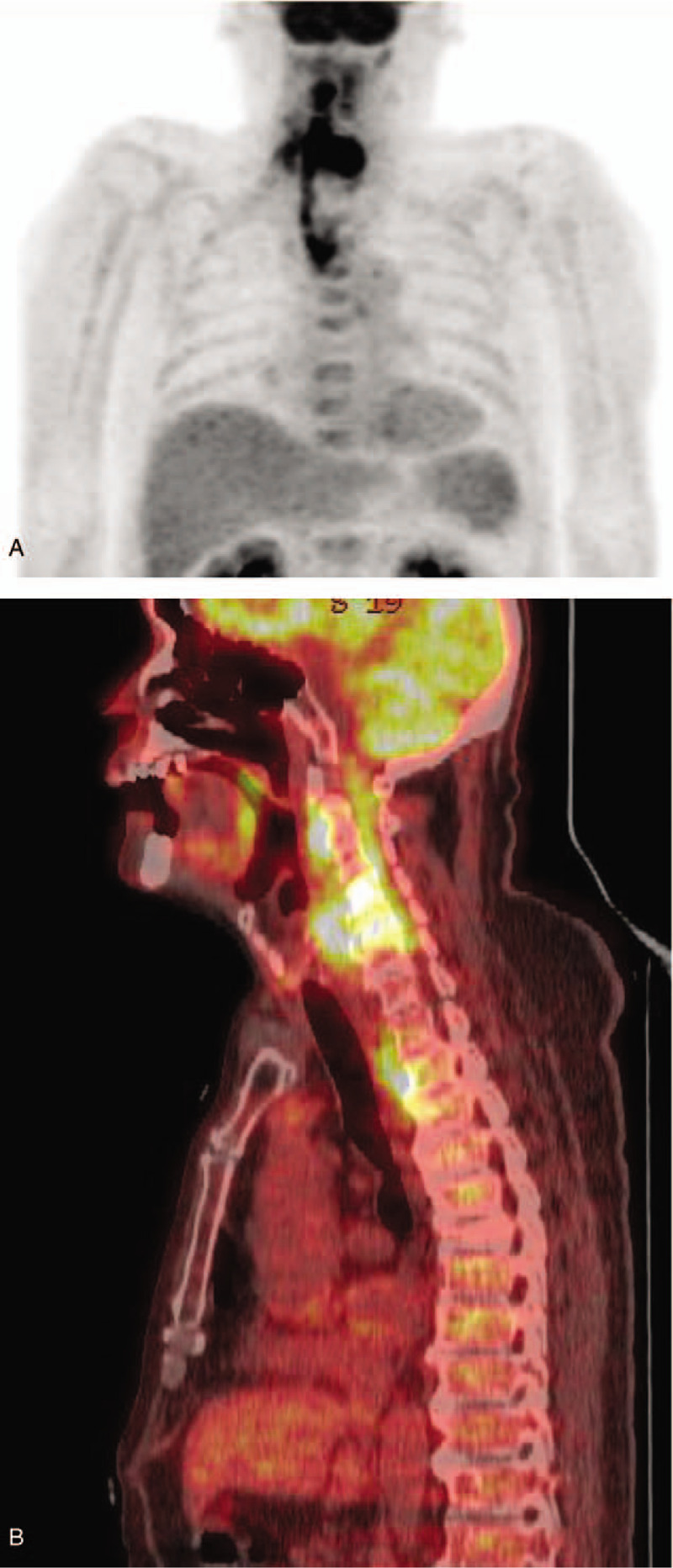
The maximum intensity projection image of positron emission tomography (A) and sagittal scan of positron emission tomography/computed tomography fusion imaging (B) reveal continuous hypermetabolic lesions along the prevertebral and danger spaces of the neck from the C2–T4 levels with associated osteomyelitis of the vertebral bodies of C4 and C5.

FDG PET/CT revealed hypermetabolic lesions in the retropharyngeal space with a maximum standardized uptake value (SUVmax) of 9.8, involving the vertebral bodies (VBs) of the C4 and C5 vertebrae and prevertebral spaces of the T1–T4 vertebrae (Fig. 1).

**Figure 2:**
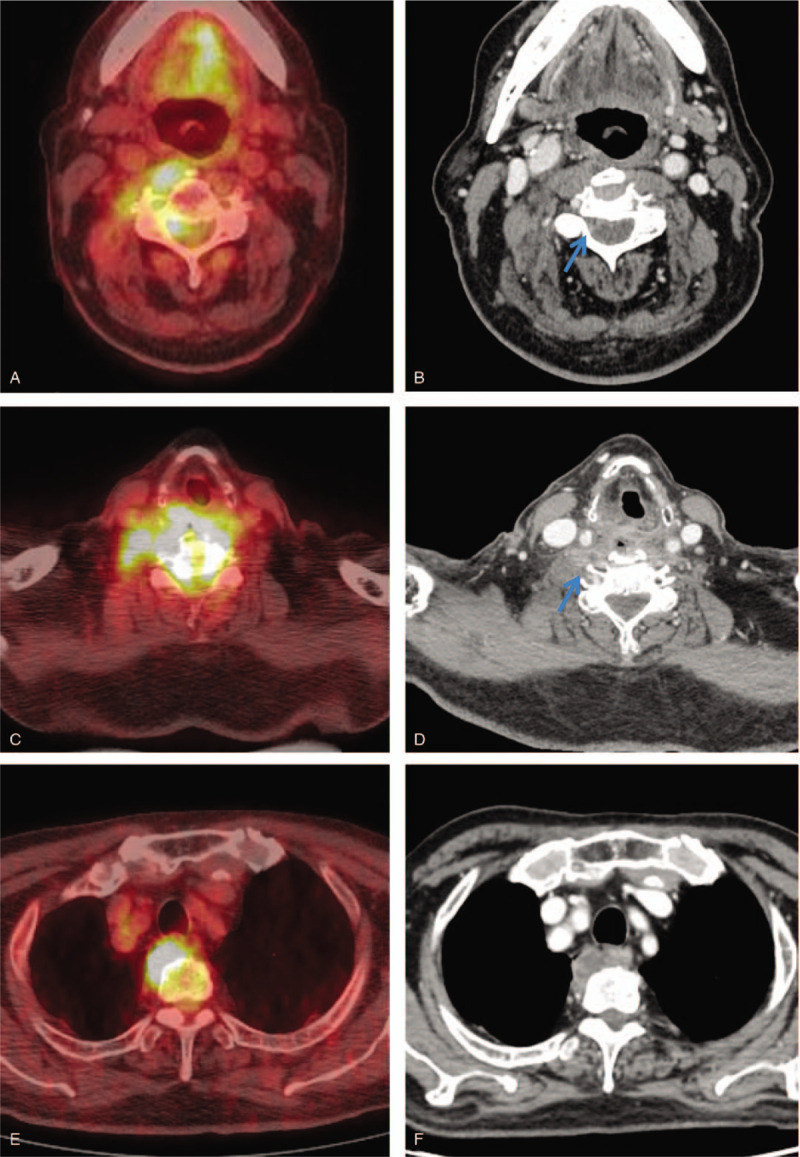
Axial images of ^18^F-fluorodeoxyglucose positron emission tomography/computed tomography (FDG PET/CT) (A, C, and E) and contrast-enhanced CT (B, D, and F) reveal hypermetabolic lesions with mild contrast enhancement along the danger and right paravertebral spaces corresponding to deep neck infections (A, B, C, and D). PET/CT reveals related osteomyelitis of the vertebral body of C4 with intense FDG uptake without significant bone changes on CT (C and D). Hypermetabolic lesions along the nerve root and spinal canal are also seen with mild contrast enhancement on CT (B and D, blue arrow). Continuous necrotizing mediastinitis with intense FDG uptake is observed along the prevertebral space of the upper mediastinum. On axial CT, central necrotic material is observed as a low-attenuation lesion (E and F).

PET/CT suggested DNI with DNM, combined with osteomyelitis of VBs of C4 and C5, and less likely, bone metastases in VBs of C4 and C5. Contrast-enhanced neck CT revealed localized abscesses with the fluid collection and rim enhancement in the retropharyngeal space and T1-T4 prevertebral space (Fig. 2). *Klebsiella pneumoniae* was isolated in the blood and sputum cultures of the patient. From clinical presentations, radiological findings and FDG PET/CT findings, the patient was diagnosed as DNM and osteomyelitis of VBs of C4 and C5, in addition to a deep neck abscess.^[3,4]^ Because of his underlying condition, a massive antibiotic therapy with ceftriaxone and ciprofloxacin was started before the surgical intervention. However, sepsis and multiorgan failure developed sequentially, and the patient died 2 weeks after the diagnosis.

## 3 Discussion

DNIs affect the fascial spaces of the head and neck, usually originating from dental or pharyngotonsillar disease. Despite the improved dental care and widespread use of antibiotics, DNIs are often encountered.

Complications of DNI, such as DNM, airway obstruction, septic shock, cervical vertebral osteomyelitis, abscess formation, and disseminated intravascular coagulopathy^[5,6]^, develop especially in old patients with predisposing comorbidities, such as diabetes mellitus, renal disease, immunocompromised status, or alcoholism.^[7–10]^ Patients usually die from sepsis with multiorgan failure.

DNM is rare, but it the most serious life-threatening complication of DNI with a frequency of approximately 3%.^[11–13]^ It is a primary complication of DNI that directly spreads to the mediastinum through the cervical and mediastinal fascial spaces. Symptoms of DNM are ambiguous, such as dysphagia, dyspnea, and restricted neck movements. The mortality rate of DNI patients with DNM can reach 20% to 50%.^[6,7,13]^ Delayed diagnosis and management could cause the high mortality associated with DNM. The criterion standard diagnostic modality for DNM is contrast-enhanced neck or chest CT.

FDG PET/CT is highly sensitive to septic or aseptic inflammation and malignant processes.^[14]^ With the expanding clinical use of PET/CT, infections are frequently encountered in the oncology work-up. Because of the longitudinal disease spread patterns and prevertebral FDG uptake pattern, DNM can be confused with the physiologic FDG uptake of the longus capitis muscle or brown fat uptake at an early stage of the disease. In our case, as an acute infection, DNM showed more severe uptake relative to the average SUVmax of reference brown fat (9.8 vs 2.4–7.0)^[15,16]^, showed continuous patterns of hyper metabolic lesions from the retropharyngeal space to the prevertebral space (Fig. 1), and showed the prevertebral uptake pattern compared to the paravertebral uptake pattern of brown fat (Fig. 2). Other possible cause of prevertebral or mediastinal hypermetabolic lesions includes tuberculosis involvement, lymphoma, mediastinal desmoid tumor, lung abscess, mediastinal metastasis of lung cancer, atypical sarcoidosis, and Castleman disease.^[17]^

In this case, radiating pain in the upper arm seems to have originated from compression or involvement of the nerve roots associated with cervical vertebral osteomyelitis. Osteomyelitis of the cervical vertebral bodies can occur in patients with DNI through direct implantation of inflammation from the parapharyngeal or retropharyngeal abscess.^[18]^ FDG PET/CT is sensitive to early infection of the spinal osteomyelitis or discitis^[19]^ but less reliable for differentiating inflammation from malignancy. In this case, there were no lytic or sclerotic lesions in the affected vertebral body, suggesting bone metastasis, so physicians could infer that it was spinal osteomyelitis involvement rather than bone metastasis. MRI of the spine helps diagnose vertebral osteomyelitis and identify the extent of infection.

Early and aggressive surgical intervention is crucial for favorable outcomes of DNM treatment.^[20]^ Although the diagnosis of DNI with DNM based on PET/CT findings was made promptly, disease progression in our patient was too fast for appropriate surgical intervention. In future cases, an early diagnosis based on FDG PET/CT findings may enable early surgical intervention of DNI with DNM in cancer patients.

## 4 Conclusion

In conclusion, this is the first report describing the findings of FDG PET/CT in a patient with DNI and DNM and cervical vertebral osteomyelitis. Early and appropriate diagnosis of DNI with DNM based on FDG PET/CT findings is necessary in cancer patients to provide immediate management and prevent the high mortality risk of DNI with DNM.

## Author contributions

**Conceptualization:** Jin Young Yoo.

**Writing – original draft:** Jin Young Yoo, Min Young Yoo, Ki Hyeong Lee, Sung-Soo Koong.
